# Using Domain Knowledge and Data-Driven Insights for Patient Similarity Analytics

**DOI:** 10.3390/jpm11080699

**Published:** 2021-07-22

**Authors:** Ronald Wihal Oei, Hao Sen Andrew Fang, Wei-Ying Tan, Wynne Hsu, Mong-Li Lee, Ngiap-Chuan Tan

**Affiliations:** 1 Institute of Data Science, National University of Singapore, Singapore 117602, Singapore; tanweiying@nus.edu.sg (W.-Y.T.); whsu@comp.nus.edu.sg (W.H.); leeml@comp.nus.edu.sg (M.-L.L.); 2 SingHealth Polyclinics, SingHealth, Singapore 150167, Singapore; andrew.fang.h.s@singhealth.com.sg (H.S.A.F.); tan.ngiap.chuan@singhealth.com.sg (N.-C.T.); 3 School of Computing, National University of Singapore, Singapore 117417, Singapore

**Keywords:** patient similarity, distance metric learning, diabetes, hypertension, dyslipidaemia

## Abstract

Patient similarity analytics has emerged as an essential tool to identify cohorts of patients who have similar clinical characteristics to some specific patient of interest. In this study, we propose a patient similarity measure called D3K that incorporates domain knowledge and data-driven insights. Using the electronic health records (EHRs) of 169,434 patients with either diabetes, hypertension or dyslipidaemia (DHL), we construct patient feature vectors containing demographics, vital signs, laboratory test results, and prescribed medications. We discretize the variables of interest into various bins based on domain knowledge and make the patient similarity computation to be aligned with clinical guidelines. Key findings from this study are: (1) D3K outperforms baseline approaches in all seven sub-cohorts; (2) our domain knowledge-based binning strategy outperformed the traditional percentile-based binning in all seven sub-cohorts; (3) there is substantial agreement between D3K and physicians (κ = 0.746), indicating that D3K can be applied to facilitate shared decision making. This is the first study to use patient similarity analytics on a cardiometabolic syndrome-related dataset sourced from medical institutions in Singapore. We consider patient similarity among patient cohorts with the same medical conditions to develop localized models for personalized decision support to improve the outcomes of a target patient.

## 1. Introduction

Diabetes, hypertension, and dyslipidaemia (DHL) are three of the most prevalent chronic diseases. Globally, the prevalence of these three conditions is about 8.5%, 25%, and 39%, respectively [1,2,3]. These medical conditions exact a heavy burden of care. Diabetes alone was estimated to contribute USD 760 billion in global health expenditure in 2019, and this is projected to grow to USD 825 billion by 2030 and USD 845 billion by 2045 [4]. Apart from economic costs, these conditions are major risk factors for cardiovascular, kidney, foot, and eye complications, which ultimately result in poorer quality of life for patients [5]. Many studies have analyzed large populations to provide statistical summaries of an “average” patient. However, these studies are expensive, time-consuming, and often subject to selection bias [6]. Further, they may not be applicable to patients whose conditions differ from this “average” patient [7].

While improvement in clinical outcomes will continue with breakthroughs in treatment for these medical conditions [8,9,10], there is a growing trend towards more proactive and personalized medicine using patient analytics to improve patient care and clinical outcomes. This is facilitated by the digitization of patients’ data and the rapid adoption of electronic health records (EHRs). However, it remains a challenge to analyze and derive insights from the huge volume of EHR data, which are multivariate, heterogeneous, and sparse. These analyses involve finding similar patients for patient stratification [11,12,13], diagnosis prediction [14,15], medical prognosis [16,17], or treatment recommendations [18,19,20]. With patient similarity analytics, personalized models can be built based on the retrieved cohort of similar patients, thus furthering the development of personalized medicine.

Existing methods to find similar patients require computing the distances between patients using information such as demographics, diagnoses, relevant laboratory test results, and medications. These methods can be broadly classified into neighborhood-based [15,16,21] and cluster-based approaches [18,22]. For neighborhood-based approaches, Lee et al. [16] utilized a cosine similarity metric to select patients similar to index patients, while Ng et al. [21] used Mahalanobis distance and formulated the retrieval of similar patients as an optimization problem. Neighborhood-based algorithms are often constrained by their scalability when applied to high dimensional data.

Different from neighborhood-based algorithms, cluster-based approaches first group patients based on their feature similarity. A new patient is then assigned to one of the pre-defined groups that has the highest similarity score. Haas et al. [18] employed unsupervised clustering approaches with Gower similarity coefficient [23] to recommend the next treatment for patients with lung cancer. However, cluster-based algorithms often do not work well for patients with complex health conditions and co-morbidities [6].

Despite the fact that many studies had proposed their own similarity metrics belonging to these two categories, some limitations exist in the proposed approaches. First, many of the proposed approaches were only applicable to datasets with a low-level of granularity, where the datasets only consisted of limited types of variables, such as only using a series of International Classification of Diseases (ICD) codes as data input [17,21]. Moreover, most of the proposed approaches were solely based on data-driven insight. Nevertheless, the insight learnt from data may not always conform to domain knowledge.

In this study, we propose a framework to encapsulate the notion of similarity among DHL patients with different comorbidities. Our work considers different sources of information from EHRs, including demographics, vital sign, laboratory test results, and prescribed medications and their corresponding dosages. We develop a patient similarity measure called D3K, which stands for data-driven and domain knowledge; our D3K approach takes into consideration domain knowledge and data-driven insights to retrieve patients that are clinically similar to a target patient. Domain knowledge insights are incorporated into the D3K approach by binning variables and using labels provided by physicians to compute the importance of the features, whereas data-driven insights are incorporated by solving a generalized Mahalanobis measure to determine the importance of the features. Our D3K approach tries to address the two limitations mentioned above: (1) D3K is applied on datasets consisting of different types of variables, including demographics, vital signs, laboratory test results, and prescribed medications; (2) D3K incorporates both data-driven and domain knowledge insights to retrieve clinically similar patients.

## 2. Materials and Methods

This study was conducted using a real-world EHR dataset consisting of de-identified patients from seven primary care clinics in Singapore with DHL conditions between 2014 and 2015. The first visit of each patient during this period is considered the base visit. The dataset contains information about the patients’ demographics; blood pressure; laboratory test results, including low-density lipoprotein, high-density lipoprotein, triglyceride, and haemoglobin A1c levels; and prescribed medications at the base visit, as well as any macrovascular complication outcome. Ethical board approval was obtained before this study was conducted (SingHealth Centralized Institutional Review Board Reference Number: 2019/2604).

A total of 169,434 unique patients with DHL visited the clinics during this period. The mean age of patients was 64.64 ± 12.03 years old, and the proportion of males to females was 46.44% versus 53.56%. The patients also displayed a bias towards the combined medical condition of hypertension and dyslipidaemia (36.64%). The second most prevalent condition among the patients was combined diabetes, hypertension, and dyslipidaemia (31.10%), followed by dyslipidaemia (13.53%). Additionally, a total of 9412 patients (5.56%) in this study cohort developed macrovascular complications.

**Figure 1:**
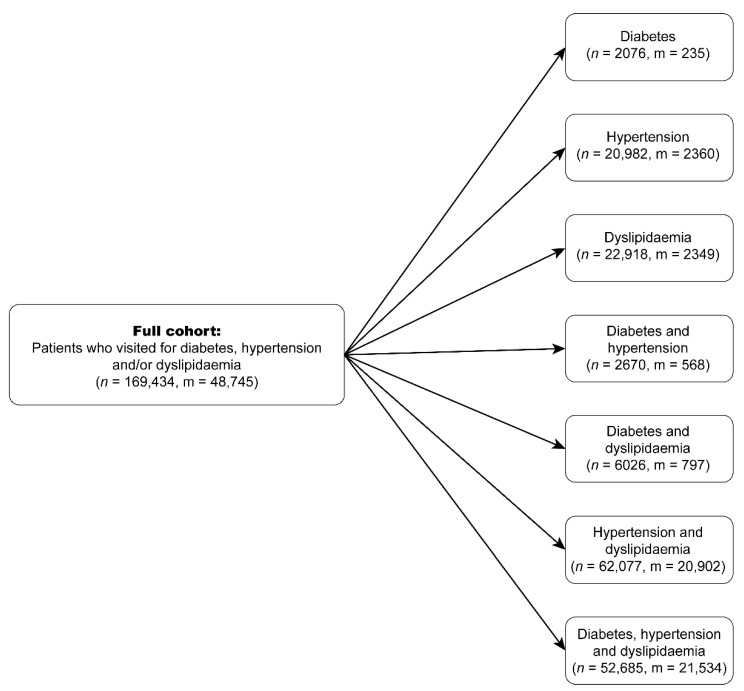
Derivation of the study cohorts (*n* denotes the number of patients and m denotes the number of patients with macrovascular complication).

Because our goal was to find similar patients having the same medical condition (DHL) or comorbidities, we partitioned the study cohort into seven different sub-cohorts, as shown in Figure 1. The baseline characteristics of the patients in each sub-cohort are shown in Table 1. The prescribed medications can be categorized into anti-diabetic, anti-hypertensive, and lipid-lowering medications. Each category was further divided into different medication classes, as shown in Table 2.

We constructed a patient profile comprising the variables listed in Table 3 as a vector in a d-dimensional feature space. We included the count of medications in each class as well as the total daily dose for each prescribed medication. This allows us to take into consideration the drug hierarchy and the disease severity. For example, patients who have been prescribed medications belonging to the same class are more similar compared to patients who have been prescribed medication belonging to different classes. Furthermore, patients with more medications are often associated with a higher disease severity level.

Our proposed patient similarity algorithm first learns a generalized Mahalanobis measure that maximizes the distance between a patient pair ($P_{i}$, $P_{k}$) who are deemed to be clinically dissimilar while minimizing the distance between patients ($P_{i}$, $P_{j}$) who are clinically similar. In other words, for each cohort, C, listed in Table 1, we have:

(1) $$\underset{W_{C}\;}{\min}\sum_{P_{i}\;\in \;C}\left(\sum_{\left(P_{i},P_{j}\right)\in similar}\sqrt{{\left(P_{i}-P_{j}\right)}^{T}\;W_{C}\;W_{C}{}^{T}\;\left(P_{i}-P_{j}\right)}-\sum_{\left(P_{i},P_{k}\right)\in dissimilar}\sqrt{{\left(P_{i}-P_{k}\right)}^{T}\;W_{C}\;W_{C}{}^{T}\;\left(P_{i}-P_{k}\right)}\right)$$

where $W_{C}$ is a transformation vector for the cohort, C, that captures the importance of the variables in the patient similarity computation.

We randomly sampled 2240 pairs of patients from the study dataset and enlisted the help of two physicians to annotate if they considered these patient pairs as clinically similar or dissimilar. Table 4 shows the statistics of the number of patient pairs in each cohort. We discarded patient pairs whom the two physicians disagreed on and used the remaining pairs to learn the vector, $W_{C}$, for each cohort, C.

However, learning the importance at the variable level is not sufficient to capture how physicians perceive patient similarity. The patient similarity computation requires a finer granularity that is value-dependent and takes into consideration the range of values of the variables. This is because what differentiates one patient from another often lies in how their vital sign and laboratory test values deviate from the normal range [24]. A patient, *P*1, with a systolic blood pressure of 150 mmHg, for example, would be more similar to a patient, *P*2, with a systolic blood pressure of 175 mmHg than to a patient, *P*3, with a systolic blood pressure of 125 mmHg. This is because, clinically, the blood pressure values indicate that patients *P*1 and *P*2 have hypertension, while patient *P*3 does not.

One common approach is to use value abstraction to convert the laboratory test values. For example, Pokharel et al. [25,26] divide the values into very low (<10th percentile), low (between 10th and 25th percentiles), normal (between 25th and 75th percentiles), high (between 75th and 90th percentiles), and very high (>90th percentile). However, this grouping does not conform to clinical practice guidelines. As a result, the retrieved patients may not be deemed similar by clinicians.

Instead, we discretized the laboratory test values into various bins based on the prevailing clinical practice guidelines [27,28,29], as shown in Table 5. This ensures that each bin corresponds to a different level of prognosis, where patients in the higher bin could have a worse prognosis compared to patients in the lower bins. Gender is a categorical variable, and we used bin 1 for females and bin 2 for males. The bins for the age and disease duration as well as medication count are shown in Table 6. In our study cohort, the minimum, median, and maximum medication counts for anti-diabetic medications, anti-hypertensive medications, and lipid-lowering medications were (0, 1, 5), (0, 1, 6), and (0, 1, 7), respectively. Further, medications belonging to HMG-CoA reductase inhibitors were discretized into three bins based on the dosage level (Table 7). Other medications were divided into three bins based on the maximum daily dose: low-intensity (≤$\frac{1}{3}$ maximum daily dose), moderate-intensity (≤$\frac{2}{3}$ maximum daily dose), and high-intensity (>$\frac{2}{3}$ maximum daily dose).

For each variable, v, we adjusted its importance in a cohort, C, and computed the score for a bin, b, as follows:

(2) $$score\left(b,\;v,\;C\right)=W_{C}\left[v\right]*\frac{b}{B}$$

where $W_{c}\left[v\right]$ is the importance of variable v and B is the total number of bins for v.

We computed the total score for a patient, P, in a cohort, C, as follows:

(3) $$total\_score\left(P\right)=\sum_{v=1}^{D}score\;\left(bin\left(\phi \left(P,\;v\right)\right),\;v,\;C\right)\;$$

where ϕ(P, v) is the value of variable v for patient P, bin(.) is the bin number that a value falls in, and D is the total number of variables.

Given two patients, P1 and P2, in the same cohort, C, we compared their scores for each variable and selected the bin with the lower score as the contributor when we computed the similarity of these two patients. This is given by:

(4) $$sim\left(P1,P2\right)=\frac{\sum_{v=1}^{D}\min\left(score\;\left(bin\left(\phi \left(P1,\;v\right)\right),\;v,\;C\right),score\;\left(bin\left(\phi \left(P2,\;v\right)\right),\;v,\;C\right)\right)}{avg\left(total\_score\left(P1\right),total\_score\left(P2\right)\right)}$$

We compared the D3K approach to retrieve similar patients in the seven sub-cohorts with the following methods:

- Euclidean distance on normalized input data.
- Locally supervised metric learning (LSML) [15]. LSML is a metric learning method to find an optimal weight vector that maximizes local class discriminability. Here, we train LSML on normalized input data with macrovascular complication as the label.

In addition, we also evaluated the performance of our approach of binning data based on the prevailing clinical guidelines and clinical understandings with binning merely based on percentiles. The percentile-based binning is given as follows: very low (<10th percentile), low (between 10th and 25th percentiles), high (between 75th and 90th percentiles), and very high (>90th percentile).

We randomly selected 10% of patients from each cohort as the test patients. For each test patient, we retrieved the top-ten similar patients and ranked them by their similarity scores. We computed the discounted cumulative gain, or *DCG*, to evaluate the effectiveness of the similarity algorithms.

For a given test patient, the *DCG* was computed as follows:

(5) $$DCG@k=\overset{k}{\underset{i=1}{\sum }}\frac{rel_{i}}{\log_{2}i}$$

where $rel_{i}$ is 1 if the ith patient in the ranked list has the same complication outcome as the test patient, or both do not have any complication. Otherwise, $rel_{i}$ is 0. Normalized *DCG*, or *nDCG*, was then computed as follows:

(6) $$nDCG@k=\frac{DCG@k}{IDCG@k}$$

where *IDCG* is ideal discounted cumulative gain computed by sorting the retrieved patients by their outcome similarities to the test patient, producing the maximum possible *DCG*. We performed this experiment 10 times and recorded the average *nDCG* for the top-ten patients in each cohort.

We also manually evaluated the patients retrieved by the D3K approach. After calculating the minimum sample size required for kappa statistic, a total of 80 patients were randomly selected from the entire study cohort as index patients for this evaluation. For each index patient, we retrieved the 10 most similar patients and another 10 random patients. We shuffled these 20 patients before presenting them to two physicians to review and evaluate which 10 patients in the list were most similar to the index patient. We analyzed the results using Cohen’s kappa and Fleiss’ kappa coefficients to determine the agreements between the physicians and D3K approach. All statistical analyses were performed using Scipy 1.4.1 library in Python 3.7 (Scotts Valley, CA, USA).

## 3. Results

### 3.1. Models Performance

**Figure 2:**
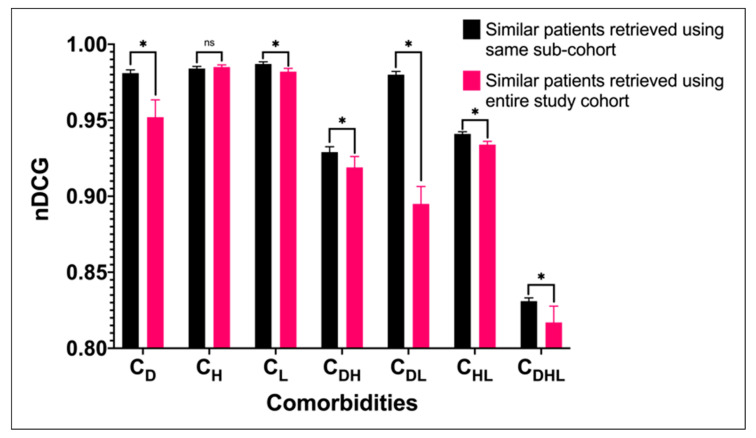
Comparison of *nDCG@10* of the D3K approach when similar patients are retrieved using sub-cohorts and entire study cohorts. * indicates statistically significant based on paired *t*-test, while ns indicates not statistically significant.

Table 8 presents the *nDCG* calculated from the top 10 similar patients retrieved by each approach. Our D3K approach achieves the highest *nDCG* in all seven cohorts, all of which are statistically significant. Figure 2 compares the results when retrieving similar patients from the entire study cohort versus from sub-cohorts of patients with the same comorbidities. Our proposed approach retrieving similar patients from sub-cohorts generally gives higher *nDCG@10*.

**Figure 3:**
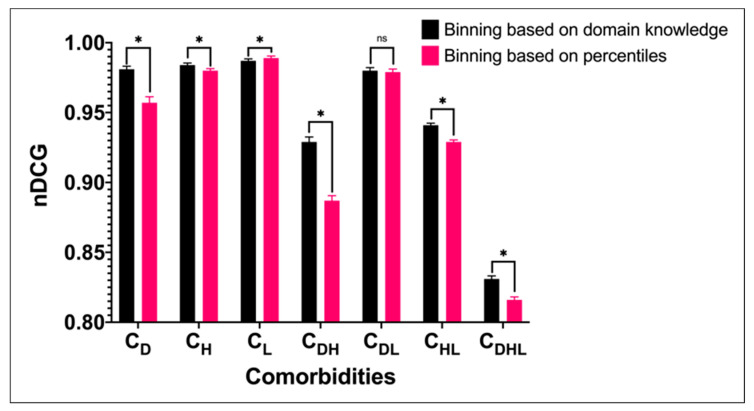
Effect of binning strategies on *nDCG@10*. * indicates statistically significant based on paired *t*-test, while ns indicates not statistically significant.

Two strategies for binning variable values are also compared. Our proposed method, which discretizes variables based on current clinical guidelines and domain knowledge, is compared to methods that discretize variables based on the 10th, 25th, 75th, and 90th percentiles. Figure 3 shows that, in general, our clinical guidelines binning strategy performs better than the percentile binning strategy, except for the C_L_ cohort.

Table 9 shows a specific patient example and the retrieved 10 most similar patients from the three approaches. The index patient was a patient with 3 years of hypertension, a systolic blood pressure of 132 mmHg, and a diastolic blood pressure of 78. As can be seen from the table, the D3K approach is not only able to retrieve clinically similar patients based on the record at the base visit, but also on the macrovascular complication outcome.

### 3.2. Kappa Statistics

Table 10 shows the analysis of the agreement between the two physicians and the D3K approach. Cohen’s kappa shows substantial agreements between the proposed D3K approach and both physicians, κ = 0.715 (95% CI: 0.666–0.764, *p* < 0.001) and κ = 0.863 (95% CI: 0.814–0.911, *p* < 0.001), respectively. There is moderate agreement between the two physicians’ judgements, κ = 0.660 (95% CI: 0.611–0.709, *p* < 0.001). The Fleiss’ kappa shows significant agreement between both physicians and our D3K method, κ = 0.746 (95% CI: 0.718–0.774, *p* < 0.001). The results show that, among 80 sample patients, both the Cohen’s kappa and Fleiss’ kappa show that the kappa coefficients are all within the range of substantial agreement level [30].

## 4. Discussion

This study was aimed at developing a patient similarity measure that incorporates domain knowledge and data-driven insights to retrieve clinically similar patients to an index patient. The results indicate that incorporating domain knowledge and data-driven insights into the similarity computation is advantageous. Our D3K approach is able to retrieve patients who are not just similar to the index patients based on the variables of interest, but also in terms of complication outcome (Table 8). Compared to the results obtained when retrieving similar patients from the entire study cohort, our proposed method, which retrieves patients from sub-cohorts, gives higher *nDCG@10* (Figure 2). This suggests that comorbidities is an important consideration in similar patient retrieval. Further, we also compare two different strategies of binning the variable values. Our domain knowledge-based binning strategy performs better than the percentile binning strategy (Figure 3), except for the C_L_ sub-cohort, possibly due to the small fraction of patients with macrovascular complications in the cohort (10.25%) compared to that of the entire study cohort (28.77%). Lastly, the manual evaluation shows that there is a substantial agreement with both physicians and the D3K approach (Table 9).

To the best of our knowledge, this is the first study that uses patient similarity analytics on cardiometabolic syndrome-related datasets sourced from medical institutions in Singapore. Our dataset contains diverse types of variables, while past studies mainly worked on datasets that only included either merely diagnosis data [31] or limited demographics and physiological data without medication information [15,16,21]. Even for those studies that worked on datasets containing medication data, the medication dosage information was ignored [18,19].

Further, previous studies focused mainly on one medical condition. In contrast, our work considers patient similarity among patient cohorts with one or more medical conditions. To ensure that the model outputs are valid and consistent with clinical understandings, we also performed a blinded manual validation with domain experts, which showed significantly good agreement levels. Although the data comprised of varying sub-cohort sizes among patients with different comorbidities, we have shown that it is still feasible to develop localized models for the various populations.

## 5. Conclusions

Adopting an appropriate similarity measure is imperative to improve patient outcomes, as it focuses on the disease perturbations and treatments relevant to index patient. In this study, we have proposed a patient similarity algorithm that incorporates both domain knowledge and data-driven insights. Our proposed D3K algorithm bins the variable values based on clinical guidelines and assigns scores in accordance with the degree of similarity between patient pairs at the bin level.

Finding similar patients not only plays an important role in personalized clinical decision support but has great potential for other downstream applications to improve patient outcomes. We envision that the proposed patient similarity algorithm may serve as a personalized clinical decision tool for medical practitioners to improve the outcomes of index patients. Future work will include augmenting the dataset with patients across multiple clinic sites and considering the temporal trajectory of the patient over multiple visits.

## Figures and Tables

**Table 1:** Baseline characteristics of patients in each study cohort.

Cohort	Description	Number of Patients	Age	Gender	
				Male	Female
C_D_	Diabetes only	2076	52.70 (±13.89)	1141	935
C_H_	Hypertension only	20,982	60.84 (±13.29)	11,001	9981
C_L_	Dyslipidaemia only	22,918	50.81 (±10.55)	14,333	8585
C_DH_	Diabetes and hypertension only	2670	63.28 (±12.69)	1436	1234
C_DL_	Diabetes and dyslipidaemia only	6026	57.23 (±11.01)	2959	3067
C_HL_	Hypertension and dyslipidaemia only	62,077	67.14 (±11.23)	29,261	32,816
C_DHL_	Diabetes, hypertension, and dyslipidaemia	52,685	67.11 (±11.07)	25,319	27,366

**Table 2:** List of categories and classes of medications in the dataset.

Category	Class	Medication
Anti-diabetic	Biguanides	Metformin
	Alpha-glucosidase inhibitors	Acarbose
	Dipeptidyl peptidase 4 inhibitors	Linagliptin
	Sulfonylureas	Gliclazide, glipizide, tolbutamide
	Sodium-glucose co-transporter 2 inhibitors	Dapagliflozin, empagliflozin
	Insulin	Rapid-acting insulin, isophane insulin, insulin glargine, insulin detemir, pre-mixed insulin
Anti-hypertensive	Diuretics	Hydrochlorothiazide, co-amilozide, indapamide, spironolactone
	Beta blockers	Atenolol, bisoprolol, nifetex ^1^, propranolol
	Alpha antagonists	Prazosin
	Sympatholytics	Methyldopa
	Angiotensin-converting enzyme inhibitors	Captopril, enalapril, lisinopril, perindopril
	Angiotensin II receptor blockers	Candesartan, losartan, telmisartan, valsartan
	Calcium channel blockers	Amlodipine, nifedipine (long-acting)
	Direct vasodilators	Hydralazine
Lipid-lowering	Hydroxymethylglutaryl-CoA (HMG-CoA) reductase inhibitors	Atorvastatin, lovastatin, pravastatin, rosuvastatin, simvastatin
	Cholesterol absorption inhibitors	Ezetimibe
	Bile acid sequestrants	Cholestyramine
	Fibric acid derivatives	Fenofibrate, gemfibrozil

^1^ Contains a combination of beta-blocker (atenolol) and calcium channel blocker (nifedipine). For this study, it was treated as a beta-blocker.

**Table 3:** List of variables of interest.

Variable	Type	Description
Age (years)	Discrete	Age at base visit
Gender	Categorical	Female, Male
Disease duration	Discrete	Number of years since diagnosis of disease
Systolic BP (mmHg)	Continuous	Systolic blood pressure
Diastolic BP (mmHg)	Continuous	Diastolic blood pressure
HbA1c level (%)	Continuous	Haemoglobin A1c level
LDL level (mmol/L)	Continuous	Low-density lipoprotein level
HDL level (mmol/L)	Continuous	High-density lipoprotein level
TG level (mmol/L)	Continuous	Triglyceride level
Total daily dose per medication	Discrete	Total daily dose for each prescribed medication
Medication count per class	Discrete	Count of medication prescribed for each class
Medication count	Discrete	Total number of medications prescribed
Macrovascular complication	Discrete	Yes if patient developed macrovascular complication within five years after base visit, otherwise no

BP, blood pressure; LDL, low-density lipoprotein; HDL, high-density lipoprotein; TG, triglycerides; HbA1c, hemaglobin A1c.

**Table 4:** Statistics of patient pairs manually annotated in each cohort.

Cohort	Number of Patient Pairs Sampled	Number of Patient Pairs Deem Similar by Both Physicians	Number of Patient Pairs Deem Dissimilar by Both Physicians	Number of Patient Pairs Discarded Due to Disagreement
C_D_	120	53	59	8
C_H_	300	125	122	53
C_L_	440	164	165	111
C_DH_	100	48	48	4
C_DL_	140	65	67	8
C_HL_	820	341	352	127
C_DHL_	320	104	132	84

**Table 5:** Range of laboratory test results for each bin.

Bin	Systolic BP (mmHg)	Diastolic BP (mmHg)	LDL Level (mmol/L)	HDL Level (mmol/L)	TG Level (mmol/L)	HbA1c Level (%)
1	Normal (<130)	Normal (<85)	Optimal (<2.6)	Optimal (≥1.6)	Optimal (<1.7)	Good control (<7)
2	High-normal (130–139)	High-normal (85–89)	Desirable (2.6–3.3)	Desirable (1.3–1.5)	Desirable (1.7–2.2)	Adequate control (7.1–8.9)
3	Grade 1 hypertension (140–159)	Grade 1 hypertension (90–99)	Borderline high (3.4–4.0)	Low (1.0–1.3)	Borderline high (2.3–3.3)	Inadequate control (9.0–11.9)
4	Grade 2 hypertension (160–179)	Grade 2 hypertension (100–109)	High (4.1–4.8)	Very low (<1.0)	High (3.4–4.4)	Poor control (12.0–13.9)
5	Grade 3 hypertension (≥180)	Grade 3 hypertension (≥110)	Very high (≥4.9)		Very high (≥4.5)	Very poor control (≥14)

BP, blood pressure; LDL, low-density lipoprotein; HDL, high-density lipoprotein; TG, triglycerides; HbA1c, hemaglobin A1c.

**Table 6:** Range of age values, disease duration, and medication counts for each bin.

Bin	Age (Years)	Disease Duration (Years)	Medication Count per Class	Total Medication Count
1	≤39	1	0	0
2	40–49	2	1	1
3	50–59	3	2	2
4	60–69	4	3	3
5	≥70	≥5	≥4	≥4

**Table 7:** Bin values for medications belong to hydroxymethylglutaryl-CoA (HMG-CoA) reductase inhibitors.

Type of HMG-CoA Reductase Inhibitors	Low-Intensity	Moderate-Intensity	High-Intensity
Pravastatin	(0, 40)	>40	-
Lovastatin	(0, 40)	>40	-
Simvastatin	(0, 20)	(20, 80)	>80
Atorvastatin	(0, 10)	(10, 40)	≥40
Rosuvastatin	(0, 5)	(5, 20)	≥20

**Table 8:** Results of *nDCG@10* for the different cohorts in the format “mean (95% confidence interval)”.

Cohort	Size	Number of Patients with Macrovascular Complications	D3K	Euclidean Distance	LSML
C_D_	2076	235	0.981 (0.979–0.983) *	0.922 (0.918–0.926)	0.905 (0.896–0.914)
C_H_	20,982	2360	0.984 (0.983–0.985) *	0.928 (0.926–0.930)	0.923 (0.921–0.925)
C_L_	22,918	2349	0.987 (0.986–0.988) *	0.936 (0.934–0.938)	0.929 (0.926–0.932)
C_DH_	2670	568	0.929 (0.926–0.932) *	0.864 (0.854–0.874)	0.849 (0.844–0.854)
C_DL_	6026	797	0.980 (0.978–0.982) *	0.918 (0.914–0.922)	0.957 (0.954–0.960)
C_HL_	62,077	20,902	0.941 (0.940–0.942) *	0.851 (0.849–0.853)	0.802 (0.801–0.803)
C_DHL_	52,685	21,534	0.831 (0.829–0.833) *	0.809 (0.807–0.811)	0.781 (0.780–0.782)

* Statistically significant based on one-way analysis of variance (ANOVA) with post-hoc Tukey’s honestly significant difference (HSD) test.

**Table 9:** A patient example and the retrieved 10 most similar patients.

	Age (Years)	Gender	Disease History (Years)	Systolic BP (mmHg)	Diastolic BP (mmHg)	Medication Count	Macrovascular Complication
Index patient	76	Male	3	132	78	2	1
D3K	69	Male	3	135	72	2	1
	87	Female	3	136	66	2	0
	84	Male	2	131	66	2	1
	73	Male	3	141	70	2	0
	74	Female	3	157	59	2	1
	82	Female	3	154	61	2	0
	68	Male	3	149	74	2	0
	69	Male	3	157	59	2	0
	91	Female	3	158	75	2	0
	77	Male	3	142	75	2	0
	*nDCG*						0.886
Euclidean distance	68	Male	3	149	74	2	0
	77	Male	3	142	75	2	0
	78	Male	3	108	71	2	0
	69	Male	3	135	72	2	1
	69	Male	3	157	59	2	0
	67	Male	3	125	75	2	0
	63	Male	3	149	90	2	0
	63	Male	3	121	65	2	0
	65	Male	3	160	75	2	0
	64	Male	3	110	60	2	0
	*nDCG*						0.431
LSML	81	Female	3	171	89	1	0
	76	Female	3	160	98	4	0
	88	Female	1	194	92	1	1
	72	Male	1	223	120	1	0
	64	Female	1	138	82	1	0
	89	Male	3	169	72	1	0
	85	Female	1	172	81	2	0
	87	Male	3	140	57	3	0
	79	Female	3	140	66	3	0
	77	Female	1	156	60	3	0
	*nDCG*						0.500

**Table 10:** Agreement analysis using Cohen’s kappa.

	Cohen’s Kappa	95% Confidence Interval	*p*-Value
Physician A vs. D3K	0.715	0.666–0.764	<0.001
Physician B vs. D3K	0.863	0.814–0.911	<0.001
Physician A vs. Physician B	0.660	0.611–0.709	<0.001

## Data Availability

The datasets analyzed during the current study are not publicly available as they contain information that is sensitive to the institution. They may be made available from H.S.A.F. at andrew.fang.h.s@singhealth.com.sg on reasonable request.
